# Arginine Regulates Zygotic Genome Activation in Porcine Embryos Under Nutrition Restriction

**DOI:** 10.3389/fvets.2022.921406

**Published:** 2022-06-23

**Authors:** Tianrui Zhang, Yingying Zheng, Tianya Kuang, Lianyu Yang, Hailong Jiang, Heming Wang, Yicheng Zhao, Rui Han, Dongsheng Che

**Affiliations:** ^1^Key Laboratory of Animal Production, Product Quality and Security of Ministry of Education, Jilin Provincial Key Laboratory of Animal Nutrition and Feed Science, College of Animal Science and Technology, Jilin Agricultural University, Changchun, China; ^2^Department of Gastroenterology and Hepatology, Zhongshan Hospital, Fudan University, Shanghai, China; ^3^Shanghai Institute of Liver Diseases, Shanghai, China; ^4^College of Clinical Medicine, Changchun University of Chinese Medicine, Changchun, China

**Keywords:** arginine, embryonic development, porcine, zygotic genome activation, oxidative stress, energy metabolism, polyamine

## Abstract

Arginine has a positive effect on pre-implantation development in pigs. However, the exact mechanism by which arginine promotes embryonic development is undefined. Here, single-cell RNA sequencing technology was applied to porcine *in vivo* pre-implantation embryos from the zygote to morula stage, it was found that that the expression of arginine metabolism-related genes clearly changed from the 2-cell stage to the 4-cell stage, when zygotic genome activation (ZGA) occurs in porcine embryos. Further analysis showed that arginine metabolism-related genes are significantly correlated with key ZGA genes. To determine the function of arginine in porcine embryos during ZGA, the *in vitro* fertilization embryos were cultured in PZM-3 medium (0.12 mM arginine, Control group), a modified PZM-3 medium (0 mM arginine, Block group) and a modified PZM-3 medium supplemented with arginine (0.12 mM arginine, Block + Arg group). The results showed that the 4-cell arrest rate was significantly increased in the Block group compared to the Control group (*P* < 0.05). The 4-cell arrest rate in the Block + Arg group was significantly decreased than that in the Block group (*P* < 0.05). Meanwhile, the expression of ZGA marker genes and SIRT1 protein in 4-cell embryos was significantly decreased in the Block group compared to the Control group, and their expression was significantly increased in the Block + Arg group. In addition, we observed that the glutathione (GSH), ATP levels, and lipid droplet contents were significantly increased, and the reactive oxygen species (ROS) level was decreased in the Block + Arg group compared to the Block group. Compared with Control group, spermine content in culture medium and the mRNA expression of ornithine decarboxylase1 (ODC1) of embryos in the Block group were significantly decreased (*P* < 0.05), and those in the Block + Arg group were significantly increased compared with the Block group (*P* < 0.05). Moreover, when difluoromethylornithine (an inhibitor of ODC1) was added to the modified PZM-3 medium supplemented with arginine, the effect of arginine on ZGA was inhibited. In summary, our findings demonstrated that arginine may regulate ZGA under nutrition restriction in porcine embryos by promoting polyamine synthesis.

## Introduction

Embryo loss is a major problem in sow reproduction. Early embryonic mortality accounts for more than 60% of the total embryonic mortality, as early gestation is the most sensitive period for embryonic death (1). Early mammalian embryos rely on the nutrients in oviduct fluid and uterine fluid to maintain their development. Amino acids are components of the fluids found within the female reproductive tract and are used to meet the needs of embryonic development (2). Amino acids are now known to be involved in intermediary metabolism (as energy substrates), in signal transduction (3), in osmoregulation, and as intermediaries in numerous pathways that involve nitrogen metabolism during mammalian embryonic development (4, 5). Arginine is widely present in follicular, oviduct, and uterine fluids (6). Although its concentration is not high, it has a profound effect on germ cell generation and embryonic development (7). Early research found that arginine was required for *in vitro* growth of mouse blastocysts (8). Arginine is continuously consumed in the medium of early pig embryos *in vitro* (9). The addition of arginine to the culture of pig *in vitro*-fertilized embryos increased the blastocyst formation rate (10). These results indicate that arginine plays an important role during early embryonic development in mammals, but the underlying mechanisms remain largely unknown. Studies of porcine embryos at single cell resolution have emerged recently (11, 12). Therefore, single-cell RNA-seq technology can be applied to explore the molecular mechanism of the role of arginine in early pig embryos.

Early mammalian embryonic development is composed of a series of highly conserved, regulatable, and predictable cell division events, including fertilization, cleavage, ZGA, morula compaction and formation of the blastocyst. ZGA is a crucial process in early embryonic development (13). Early embryonic development is first controlled by maternal transcripts and proteins. Subsequently, ZGA is initiated, and maternal factors are gradually cleared. This process is referred to as the maternal-to-zygotic transition (MZT) (14). Activation of the zygotic gene expression program involves cell cycle length, transcription inhibitors and activators, changes in chromatin organization and removal of maternal material (15). Errors in these biological phenomena can hinder the activation of the zygotic genome. And there is increasing evidence that embryonic developmental arrest *in vitro* is associated with embryonic ZGA (16). The time of mammalian embryonic developmental arrest is consistent with the time of ZGA. Developmental arrest in pigs, sheep, cattle, etc. occurs in the G2 phase when ZGA occurs (17). Embryos in the G2 stage may be very sensitive to changes in the environment. During *in vitro* culture, the occurrence of ZGA was affected due to the discomfort of culture conditions. Recent studies have found that embryos were blocked at the 2-cell stage in devoid of all amino acids, proteins and pyruvate, and conclude that exogenously supplied pyruvate is essential for activation of the zygotic genome (18). Lack of ZGA is the likely cause of the cell-cycle block. Low-level pyruvate inhibits maternal mRNA clearance in mice (19). To determine the function of ACSS1/2 in porcine embryos during ZGA, the IVC medium was replaced with a modified PZM-5 (without amino acids, hypotaurine and pyruvate), and finds that ACSS2 mainly generates acetyl-CoA for histone acetylation in porcine early embryos under metabolic stress during ZGA (20). However, there is no report on the function of arginine in porcine embryos during ZGA.

L-arginine is a versatile amino acid that serves as a building block for protein synthesis and as a precursor for multiple metabolites, including polyamines, creatine, and nitric oxide (NO), which can help improve embryo development and survival (21). Arginine is used by nitric oxide synthase (NOS) enzymes to produce NO. The three isoforms of NOS include neuronal (NOS1), inducible (NOS2), and endothelial (NOS3). NO also plays an important role in the development of embryos (10). Adding the NO synthesis inhibitor L-NAME to embryo culture medium inhibited porcine, mouse and bovine embryonic development and reduced the ability of cleaved embryos to progress to the blastocyst stage. NOS3 may play a major role in pig embryos (10). In addition, some studies have shown that adding polyamines to the culture medium of porcine parthenogenetically activated embryos significantly increases the blastocyst formation rate compared with the control group, and finds that the expression of apoptosis-related genes decreases, thus suggesting that polyamines can improve blastocyst formation rate by inhibiting early embryonic apoptosis (22). Polyamines are modulators of embryonic arrest, and that polyamine deficiency can lead to developmental arrest (23, 24).

In this study, arginine metabolism-related genes were first observed to be expressed during ZGA in porcine embryos. Further analysis found a correlation between arginine metabolism and ZGA. Therefore, we speculate that arginine affects ZGA. Then, the effect of arginine on ZGA was investigated under nutrition restriction conditions. Difluoromethylornithine (DFMO) was used to explore the metabolic pathways by which arginine functions during ZGA. The results revealed that arginine may regulate ZGA under nutrition restriction conditions by promoting polyamine synthesis.

## Materials and Methods

### Chemicals

Unless otherwise stated, all chemicals and reagents used in this study were purchased from Sigma–Aldrich Chemical Company (St. Louis, MO, United States).

### Collection of Pre-implantation Embryos *in vivo*

The *in vivo* embryos were derived from multiparous northeast min pig sows (*n* = 10). The sows were naturally mated with the same boar after natural estrus. After 2–3 natural matings (interval 12 h), surgery was performed as shown previously (25). Briefly, the pigs were anesthetized with 1.5–2.5 mg/kg propofol (Xi'an Libang Pharmaceutical Co. Ltd., Shaanxi, China) intravenously and maintained with 3% isoflurane (Shanghai Abbott Pharmaceutical Co. Ltd., Shanghai, China). Using abdominal surgery, the embryos are flushed from the oviduct or uterus to obtain embryos at five developmental stages, as follows: Zygote (obvious sperm on the zona pellucida of the embryo; the second polar body is used for sequencing), 72 h after estrus (after standing reflex); 2-cell stage, 84–90 h after estrus; 4-cell stage, 96–102 h after estrus; 8-cell stage, 114–120 h after estrus; and morula stage, 150–156 h after estrus (Table 1). Embryos with good quality, good shape, and uniform blastomeres at the corresponding developmental stages were screened out under a microscope. Embryos were recovered at 38.5°C for 2 h before single blastomere isolation. After the operation, analgesia and anti-infective treatment can be given according to the condition of the pig (26). All procedures were approved by the Animal Care and Use Committee of the Key Laboratory of Animal Production, Product Quality and Security, Ministry of Education, in accordance with the Chinese guidelines for animal welfare and experimental protocols (Approval No. KT2015001).

**Table 1:** Number and collection time of cells for single-cell RNA-seq analysis.

**Stage**	**No. of embryos**	**No. of single cells**	Collected hours^*^
Zygote	3	3	72 h
2-cell	2	4	84–90 h
4-cell	2	4	96–102 h
8-cell	2	4	114–120 h
Morula	2	4	150–156 h
Total	11	19	

* *Collected N hours after estrus*.

### Isolation of Single Blastomeres

After obtaining embryos at each stage, the embryos with zona pellucida were transferred to 0.1 mmol/L ethylene diamine tetraacetic acid (EDTA; Ambion, Austin, TX, United States) for 20 min, and the zona pellucida was removed with an acidic solution [1% hydrochloric acid diluted in phosphate buffer solution (PBS)], followed by repeated washing with new PBS three times or more (27). The zona pellucida-free embryos were then placed in 0.05% trypsin (Gibco, Grand Island, NY, United States; diluted in PBS) for 2.5–3 min and subsequently transferred to new PBS (Gibco, Grand Island, NY, United States) droplets for repeated washing. Single blastomeres were gently isolated using a mouth pipette, and each separated single blastomere was again transferred to a new PBS droplet for repeated washing. Finally, each blastomere was transferred to 6 μl of SMART-Seq^TM^ v4 kit lysate (Clontech Takara Bio, Mountain View, CA, United States).

### Preparation of Single-Cell cDNAs, RNA-Seq Library Preparation, and Sequencing

The SMART-Seq^TM^ v4 Ultra^TM^ Low Input RNA Kit for Sequencing Clontech (Takara, Mountain View, CA, United States) was used for embryonic single cell lysis and first-strand cDNA synthesis (total RNA samples were stored in RNase-free water for direct synthesis of first-strand cDNA) (28). Then, full-length LD-PCR amplification of the first-strand cDNA was carried out, and the amplified double-stranded cDNA (ds-cDNA) was purified by an AMPure XP system (Beckman Coulter, Beverly, CA, United States). Qubit (Invitrogen, Carlsbad, CA, United States) was used for quantitative detection of ds-cDNA. The ds-cDNA was subjected to ultrasonic disruption using the Covaris S2 system (Covaris, Inc., Woburn, MA, United States), and the broken double-stranded short fragments were subjected to end repair, polyA tailing and sequencing adapter ligation. To preferentially select cDNA fragments of ~200 bp in length, the library fragments were purified with an AMPure XP system (Beckman Coulter, Beverly, CA, United States), and PCR was performed. The library quality was assessed on the Agilent Bioanalyzer 2100 system (Agilent Technologies, Santa Clara, CA, United States). After confirming the quality, different libraries were pooled to achieve the effective concentration and target fragment size required for HiSeq sequencing.

### Mapping and Gene Expression Analysis

Hisat2 v2.0.4 software (29) was used for genomic mapping of the filtered sequence. HISAT can effectively map the spliced reads in the RNA sequencing data. In the analysis, the mismatch parameter was set to 2; other parameters were set to the software default values. HTSeq v0.9.1 software (30) was used to estimate gene expression levels by counting the number of sequencing reads that were mapped to the genomic region or exon region of the porcine genome (Sscrofa10.2.90), and the union model was used. The read count is positively related to both gene length and sequencing depth, in addition to being proportional to the true expression level of the gene. To eliminate this technical bias, gene expression levels were expressed in FPKM (expected number of fragments per kilo base of transcript sequence per million base pairs sequenced) (31). An FPKM value >0.1 was considered to indicate gene expression. Differential expression analysis of the two conditions was performed using the DEGseq R package (1.20.0) (32). The *P*-Values were adjusted using the Benjamini & Hochberg method. A corrected *P*-Value of 0.005 and log2 (fold change) of one were set as the thresholds for significant differential expression. We performed hierarchical clustering analysis of the FPKM of differential gene expression, and we also used the K-means method to cluster the relative expression level log2 (ratio) of the differentially expressed genes. The clustering algorithm divides the differentially expressed genes into several clusters. Genes in the same cluster have similar trends in expression levels under different experimental conditions. The Cufflinks v2.1.1 Reference Annotation Based Transcript (RABT) assembly method was used to construct and identify both known and novel transcripts from TopHat alignment results (33). Principal component analysis (PCA), cluster analysis, and heatmap calculation and drawing were performed using the R software version 3.2.2. We used KOBAS software (34) to test the statistical enrichment of differentially expressed genes and genomically activated genes in KEGG pathways. Pearson's correlation was applied for correlation analysis (35).

### Porcine Oocyte Collection and *in vitro* Maturation (IVM), Fertilization (IVF), and Culture (IVC) of Porcine Embryos

Porcine ovaries were obtained from local slaughterhouses and transferred to 0.9% NaCl at 38°C in the laboratory within 1 h. Thereafter, follicular fluids were collected from 3 to 6 mm antral follicles using a 10 ml disposable syringe and an 18-gauge needle. The fluids were then collected in a 15 ml conical tube. The debris was allowed to settle at 38°C. The supernatants were subsequently discarded, and the precipitates were washed with PBS containing 2% fetal bovine serum (FBS). The cumulus–oocyte complexes (COCs) were then selected under a stereomicroscope. Only COCs with homogeneous cytoplasm and more than three layers of cumulus cells were selected from follicular fluids. Approximately 55–60 COCs were transferred to each well in a four-well Nunc dish (Nunc, Roskilde, Denmark). Each well contained 500 μl of maturation medium (TCM-199; Invitrogen Corporation, Carlsbad, CA, United States) supplemented with 10% porcine follicular fluid (PFF), 0.1 mg/ml cysteine, 0.91 mmol/L sodium pyruvate, 3.05 mmol/L glucose, 1 mg/ml polyvinyl alcohol (PVA), 75 mg/ml penicillin, 50 mg/ml streptomycin, 10 ng/ml epidermal growth factor, 15 IU/ml pregnant horse serum gonadotropin (PMSG), and 15 IU/ml human chorionic gonadotropin (hCG). For IVM, selected COCs were matured for 42–44 h at 38.5°C and a 5% CO_2_ atmosphere. At 44 h after IVM, cumulus cells were removed with 0.1% hyaluronidase in M199-HEPES (Invitrogen, Corporation, Carlsbad, CA, United States), and metaphase II (MII) oocytes with extruded first polar bodies were selected for IVF. The oocytes were washed three times with modified Tris-buffered medium (mTBM) (36) and placed into 50 μl drops of mTBM covered with mineral oil in a 60 mm Petri dish. Spermatozoa were resuspended in mTBM (2.5–5 × 10^7^ spermatozoa per ml). Oocytes were coincubated with spermatozoa for 5–6 h at 38.5°C and 5% CO_2_. To explore the effect of arginine on porcine ZGA, modified PZM-3 medium was developed (20) [108 mmol/L NaCl, 10 mmol/L KCl, 0.35 mmol/L KH_2_PO_4_, 0.40 mmol/L MgSO_4_· 7H_2_O, 25 mmol/L NaHCO_3_, 2 mmol/L Ca-(lactate)_2_·5H_2_O]. Presumed zygotes were cultured in 500 μl of PZM-3 (37) medium (Control group), modified PZM-3 medium (called Block group) or modified PZM-3 medium supplemented with 0.12 mmol/L arginine (called Block + Arg group). To analyze the metabolic pathways in which arginine is involved in ZGA, zygotes were cultured in modified PZM-3 medium supplemented with 0.12 mmol/L arginine (Block + Arg) or modified PZM-3 medium supplemented with ODC1 inhibitor [difluoromethylornithine, DFMO; S4582; 2 mmol/L (38); Selleck, Houston, TX, United States] and 0.12 mmol/L arginine (Block + Arg + DFMO). The 4-cell arrest rate was determined at 72 h after IVC as the ratio of the number of embryos arrested the 4-cell stage to the total number of embryos. The experiments were repeated three to five times.

### Measurement of Intracellular GSH and ROS Levels

The GSH and ROS levels of 4-cell embryos were measured as shown previously (39). Briefly, GSH in the cytoplasm was stained using Cell Tracker Blue (4-chloromethyl-6,8-difluoro-7-hydroxycoumarin; 10 mmol/L; Invitrogen, Carlsbad, CA, United States), and ROS were stained using H_2_DCFDA (20,70-dichlorodihydrofluorescein diacetate; 10 mmol/L; Invitrogen, Carlsbad, CA, United States). The 4-cell embryos from each group were selected and incubated in the dark in PBS-PVA supplemented with 10 mmol/L Cell Tracker Blue or 10 mmol/L H_2_DCFDA for 30 min. The 4-cell embryos were then washed with fresh PBS-PVA and transferred to a 100 μl drop of culture medium for fluorescence measurement using an epifluorescence microscope (EVOS™ FL Auto; Life).

### ATP Assay

Denuded 4-cell embryos were washed three times in PBS-PVA, fixed with 4% paraformaldehyde-PBS for 1 h, washed three times, and then incubated in PBS-PVA supplemented with 500 nmol/L BODIPY ^FL^ ATP (A12410; Molecular Probes, Eugene, OR, United States) for 1 h at room temperature in the dark (40). Four-cell embryos were washed three times in PBS-PVA and captured using an epifluorescence microscope (EVOS™ FL Auto; Life).

### Lipid Droplet Staining

BODIPY 493/503 (D3922; BODIPY-LD; Molecular Probes, Eugene, OR, United States) is a neutral lipid dye that has been used to detect lipid droplets in 4-cell embryos (40). The 4-cell embryos were fixed in 4% paraformaldehyde–PBS for 1 h at room temperature and washed in PBS. Then, the embryos were permeabilized in 0.5% Triton-X100 at room temperature for 1 h. The 4-cell embryos were washed in PBS before being stained in BODIPY-LD that was prepared by dissolving 10 mg BODIPY-LD in absolute DMSO to a concentration of 2.5 mg/ml and further diluted to a final concentration of 10 μg/ml in PBS with 1% PVA to prevent adhesion between the denuded 4-cell embryo and the pipettes or dishes. Then, the 4-cell embryos were stained for 1 h at room temperature in the dark, washed three times in PBS and mounted on cover slips. Images of each 4-cell embryo were captured using an epifluorescence microscope (EVOS™ FL Auto; Life).

### Quantification of Polyamine in the Media

At 48 h after IVC, the culture medium was collected, and its polyamine content was measured. The polyamine content was quantified by UHPLC–MS/MS as previously described (41). Briefly, 50 μl samples were precisely transferred to a 1.5 ml EP tube. After the addition of 200 μl acetonitrile, the clear supernatant was prepared by ultrasound, standing, and centrifugation. A 100 μl aliquot of the clear supernatant (or standard solution) was transferred to an Eppendorf tube, incubated at 4°C for 1 h in the dark, and then mixed with 50 μl of 20 mg/ml dansyl chloride in acetonitrile and 50 μl of 0.1 mol/L sodium bicarbonate. Dansyl derivatives were added to 50 μl of 0.1% formic acid in water and vortexed for 15 s, followed by centrifugation at 12,000 rpm and 4°C for 10 min. An 80 μl aliquot of the clear supernatant was transferred to an autosampler vial for UHPLC-QQQ-MS (Agilent, 1290UHPLC-6460MS, Santa Clara, CA United States) analysis.

### Measurement of Intracellular NO Content

The NO content of the 4-cell embryos was stained using DAF-FM-DA (10) (4-amino-5-methylamino-2′,7′-difluorofluorescein diacetate, Life Technologies, Carlsbad, CA, United States). The 4-cell embryos from each treatment group were washed with PBS-PVA and transferred to wells containing 500 μl of PBS-PVA plus 10 μmol/L DAF-FM-DA. Embryos were incubated in DAF-FM-DA for 40 min, then washed in PBS-PVA, and finally subjected to fluorescence measurement using an epifluorescence microscope (EVOS™ FL Auto; Life).

### Analysis of Gene Expression by Quantitative Real-Time PCR

The mRNA was extracted from 100 embryos of each group using the Micro RNA Extraction Kit (QIAGEN, RNeasy Mini Kit, Hilden, Germany) according to the manufacturer's instructions. The PrimeScriptTM RT Reagent kit (Takara, Kusatsu, Japan) was used for reverse transcription to generate cDNA. Quantitative real-time PCR (qRT-PCR) was performed using SYBR premix ExTaqTM (Takara, Kusatsu, Japan) and an ABI Step One Plus real-time PCR system. The reaction parameters were 95°C for 30 s, followed by 40 two-step cycles of 95°C for 5 s and 60°C for 30 s. All primers used for PCR amplification are shown in the Table 2. The experimental setup consisted of three replicate wells in the same batch, and the expression level of each gene was calculated according to the 2^ΔΔCT^ method from the Cq values of the three replicate wells of the same sample, with the 18S rRNA Cq value of the same sample used as the internal reference (39).

**Table 2:** Primers list for qRT-PCR.

**Gene name**	**Genebank number**	**Primer sequence, 5^**′**^-3^**′**^**	**Size,bp**
RN18s	NW_018085108.1	F: CCCACGGAATCGAGAAAGAG	122
		R: TTGACGGAAGGGCACCA	
EIF1A	NM_001243218.1	F: GGTGTTCAAAGAAGATGGGCAAGAG	115
		R: TTTCCCTCTGATGTGACATAACCTC	
DPPA2	XM_003358822.4	F: CCGTTCCTGCTTCTGTTGAGACC	105
		R: GGCGAACCCAACCTTCTGTATCTG	
ZSCAN4	XM_021097584.1	F: GCCCAGAAAGTCTTCCCATGTGAG	94
		R: GCCTCTCATCATTGTGTCTCCTCTG	
DNMT1	NM_001032355.1	F: CCCTGGCAAACGGAAACCTGAG	122
		R: CGGCAACTGAGTCTCTGGATGTAAC	
ODC1	NM_001122983.1	F: TTTGGAGCGGGCGAGGGATC	119
		R: CGAAGACACAGCGGGCATCAG	
NOS3	NM_214295.1	F: AGGCTCTCACCTTCTTCCTGGAC	95
		R: CTGCTGTTCGCTGGGCTCTTC	
GLUD1	NM_001244501.2	F: TCGTGGAGGACAAGCTAGTGGAG	119
		R: GGACAGGCTCAGCACATGGTTG	
OAT	NM_001185141.1	F: GGGTGGAGGCTGGAGAGACTG	142
		R: GTGGAACTGGAGATCGCAGACAAC	
ASS1	XM_003353686.4	F: CTGCATCCTCGTGTGGCTGAAG	94
		R: CTTCCTGGCTTCCTCAAAGTCTTCC	

### Immunofluorescence Staining

The zygotes of each group were cultured for 48 h, and 4-cell embryos were collected. After removing the zona pellucida, they were moved to PBS-PVA, washed 2–3 times, and fixed in 4% PBS-paraformaldehyde for 30 min. Then, the embryos were permeabilized in 1% Triton X-100 at room temperature for 1 h. After three washes in PBS/PVP for 5 min each, the samples were blocked in 2% BSA for 1 h. Rabbit monoclonal SIRT1 antibody (A0230; ABclonal, Wuhan, China; 1:200) was applied overnight at 4°C. After three washes in washing buffer, the embryos were incubated with Alexa Fluor 488-conjugated goat anti-rabbit IgG (A-11001; Invitrogen, Carlsbad, CA, United States; 1:400) at room temperature for 1 h. After three washes, the embryos were incubated for 5 min with Hoechst 33342 dye (R37605; Invitrogen, Carlsbad, CA, United States; 5 μg/ml) prepared in PBS-PVA. Negative controls were performed by omitting specific antibodies. Finally, the embryos were mounted on glass slides and examined using an epifluorescence microscope (EVOS™ FL Auto; Life).

### Statistical Analysis

Data are expressed as the mean ± standard error. The fluorescence intensity of the embryos was analyzed using ImageJ software (National Institutes of Health, Bethesda, MD, United States). Each experiment was performed in triplicate. Means were compared by one-way analysis of variance followed by Duncan's multiple range test using SPSS software (SPSS, Chicago, IL, United States). Differences were considered significant when *P* < 0.05.

## Results

### Single-Cell Transcriptome Analysis Reveals *in vivo* Transcriptional Patterns of Arginine Metabolism-Related Genes During Early Embryonic Development in Pigs

**Figure 1:**
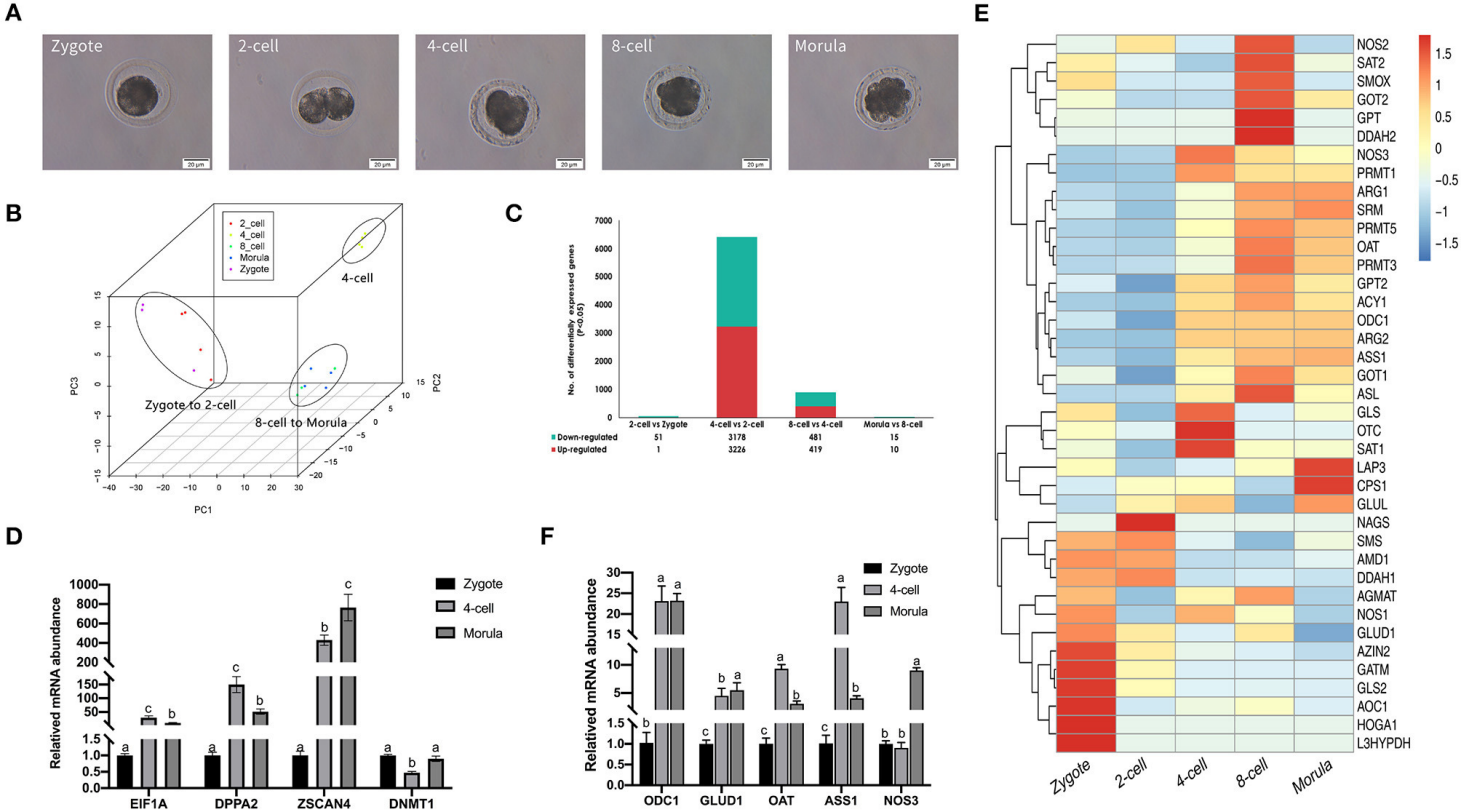
Gene expression landscape of porcine pre-implantation development. **(A)** Microscopic imaging of porcine pre-implantation embryos *in vivo* (from left to right: zygote, 2-cell, 4-cell, 8-cell, and morula). **(B)** PCA of the transcriptome of single blastomeres from five stages of an *in vivo* porcine pre-implantation embryo. Dots of the same color represent blastomeres from different embryos at the same developmental stage. **(C)** Histogram showing upregulated and downregulated genes between two adjacent stages. **(D)** Results of qRT-PCR analysis of the expression of key zygotic genes. *n* = 3; different letters (a, b) indicate significant differences (*P* = 0.05). **(E)** Results of qRT-PCR analysis of the expression of arginine metabolism-related genes. *n* = 3; different letters (a, b) indicate significant differences (*P* = 0.05). **(F)** Arginine metabolism in porcine pre-implantation embryos. Arginine metabolism-related pathways from the KEGG database are shown in hierarchical clusters at five embryonic developmental stages.

Single-cell transcriptome analysis was performed on 19 samples collected at five early embryonic development stages (zygote, 2-cell, 4-cell, 8-cell, and morula stages) from Northeast Min pigs (Figure 1A). We first analyzed how many genes were expressed in each of the 19 embryonic blastomeres. There were 12,171 genes with fragments per kilo base per million (FPKM) values >1 in at least one set of samples, accounting for 32% of the total reference genes (38,198). To determine whether the gene expression profiles varied by embryonic development stage, we analyzed the RNA-seq data of the blastomeres by PCA, which divided the samples into three major groups (Figure 1B). One of the groups contained zygotes and 2-cell blastomeres, while 4-cell, 8-cell and morula blastomeres segregated into different groups.

Differential gene expression analysis also supported this finding (Figure 1C). The number of differentially expressed genes between the 2-cell and zygotic stages was very small, at only 52. The number of differentially expressed genes between the 2-cell and 4-cell stages was the highest, reaching 6,404. We found that 3,226 genes were significantly upregulated between the 2-cell and 4-cell stages [log2 fold change (4-cell/2-cell) > 0, *P* < 0.001], 3,178 genes were significantly downregulated [log2 fold change (4-cell/2-cell) < 0, *P* < 0.001], and the rate of change was most obvious. The levels of transcripts at the 4-cell stage were significantly increased. Another significant difference occurred between the 4-cell and 8-cell stages. There were 900 differentially expressed genes between these stages, of which 419 genes were upregulated and 481 genes were downregulated in the 8-cell stage. The 8-cell and morula stages clustered together, and the number of differentially expressed genes was also small, at only 25. To confirm the high-throughput results from RNA-seq, we performed qRT-PCR on zygotic genes using IVF embryos at the zygote, 4-cell and morula stages (*n* = 3). Among the selected genes were key ZGA genes, including *EIF1A, DPPA2, ZSCAN4*, and *DNMT1* (Figure 1D). The qRT-PCR results substantiated those of RNA-seq, showing that these key ZGA genes all began to be highly expressed during the 4-cell stage. These data indicate that the major porcine ZGA occurs in the 4-cell stage.

During porcine pre-implantation embryonic development, genes associated with arginine metabolism were active at the 4-cell stage, and their expression showed an upward trend (Figure 1E). We also tested the expression of major arginine metabolism-related genes with qRT-PCR (Figure 1F). The variation tendency in these genes was similar to that found in the transcriptome data. The results suggest that arginine metabolism plays a key role in regulating the development of porcine embryos.

### Correlation of Arginine Metabolism and Porcine ZGA

**Figure 2:**
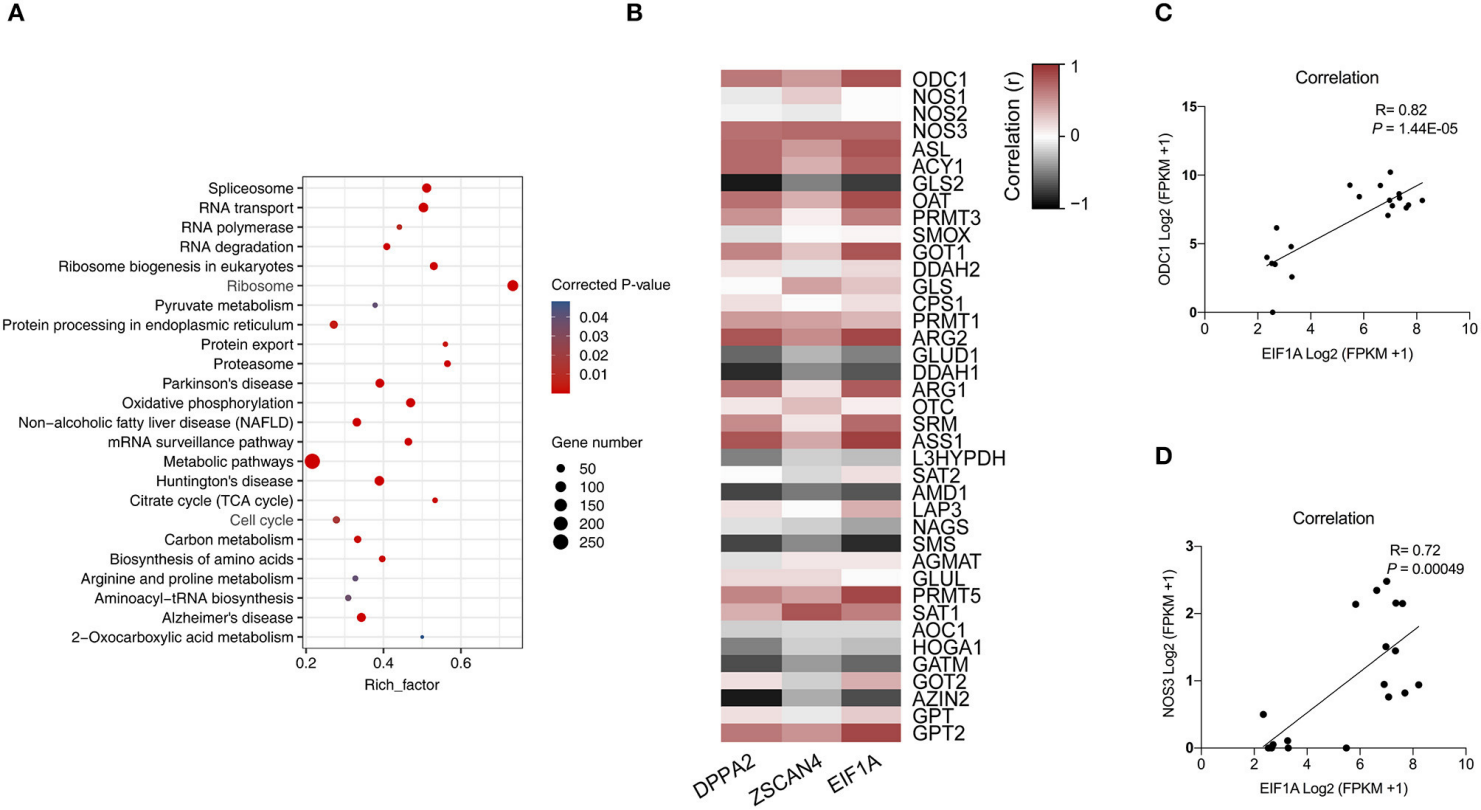
Correlation analysis of arginine metabolism and ZGA. **(A)** This scatter plot shows the KEGG pathways that were significantly enriched among the differentially upregulated genes between 4-cell and 2-cell stage (3,226). **(B)** Heatmap of correlation of arginine metabolism-related genes and key ZGA genes. Red represents a positive correlation, and black represents a negative correlation. The closer the *R* value is to 1, the darker the color. **(C)** Scatter plot of the correlation between *ODC1* gene and *EIF1A* gene expression. **(D)** Scatter plot of the correlation between *NOS3* gene and *EIF1A* gene expression.

KEGG enrichment analysis of 3,226 genes that were upregulated between the 4-cell and 2-cell stages revealed more than 200 pathways, of which 24 were significantly enriched (*P* < 0.05) (Figure 2A), including RNA transport, spliceosome, protein export, and other transcriptional and translational pathways. The metabolic pathways contained the most genes (252 genes). Interestingly, oxidative phosphorylation, amino acid biosynthesis, carbon metabolism, citric acid cycle (TCA cycle), pyruvate metabolism, arginine and proline metabolism, and 2-oxocarboxylic acid metabolism pathways are critical for ZGA.

To elaborate the relationship between arginine metabolism and ZGA, we analyzed the correlations among arginine metabolism-related genes and key ZGA genes, such as *EIF1A, DPPA2*, and *ZSCAN4* (Figure 2B). The results showed that 25 of 39 arginine metabolism genes were significantly correlated with *EIF1A, DPPA2*, and *ZSCAN4* (*P* < 0.05), among which the *ODC1* gene (*R* = 0.82, *P* = 1.44E-05) (Figure 2C) and *NOS3* gene (*R* = 0.72, *P* = 0.00049) (Figure 2D) were significantly correlated with the *EIF1A* gene. This shows that there was a correlation between arginine metabolism and ZGA. Further experiments are needed to validate this analysis.

### Arginine Promotes ZGA Under Nutrition Restriction

**Figure 3:**
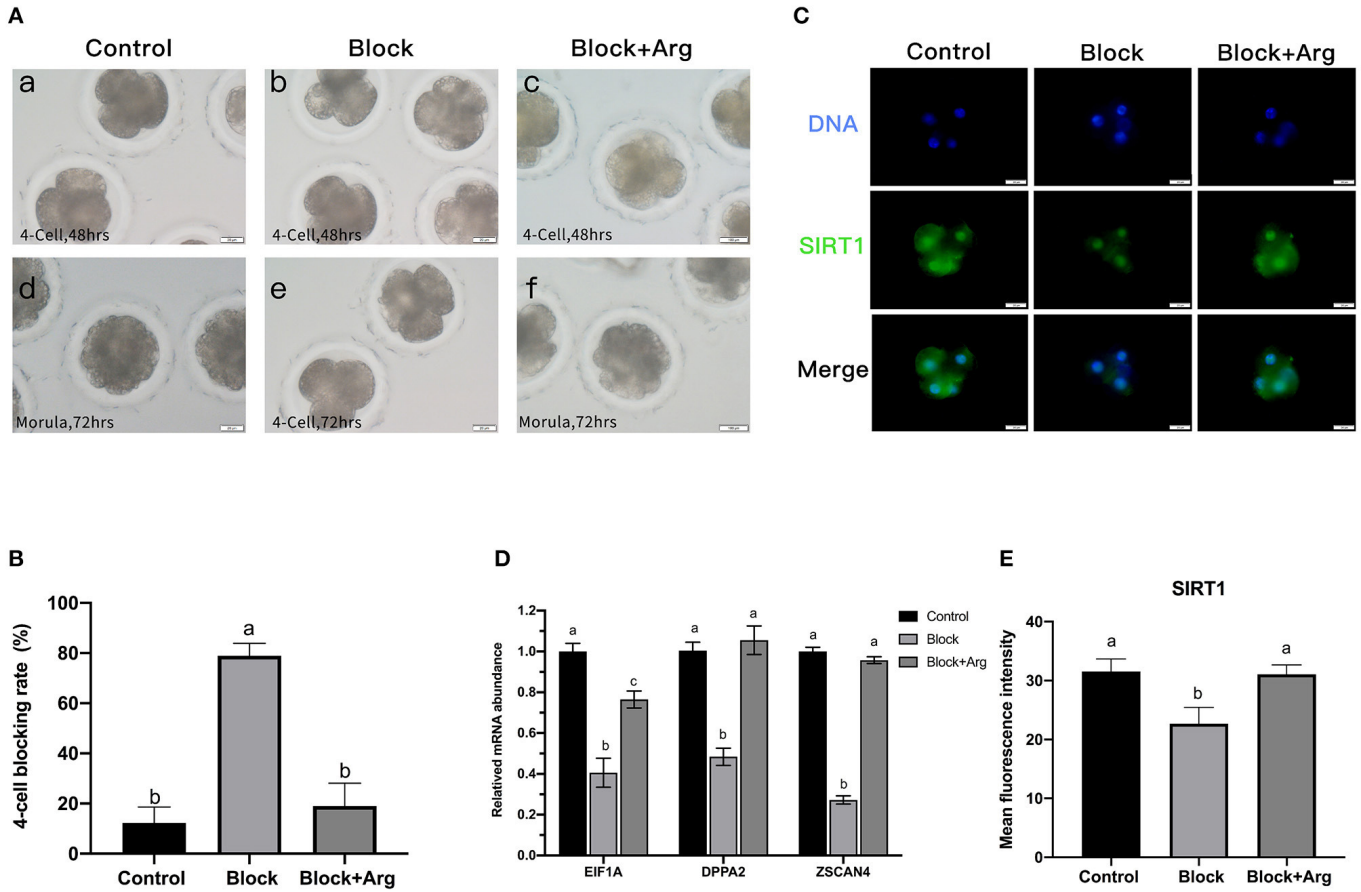
Effects of arginine on the 4-cell block and ZGA under nutrition restriction. **(A)** Developmental status of embryos cultured in different media. Embryos cultured in PZM-3 medium as a Control group at (a) 48 h (4-cell) and (d) 72 h (morula). Embryos cultured in mPZM-3 medium as a Block group at (b) 48 h (4-cell) and (e) 72 h (4-cell block). Embryos cultured in mPZM-3 medium supplemented with arginine as the Block + Arg group at (c) 48 h (4-cell) and (f) 72 h (morula). **(B)** The 4-cell arrest rate at 72 h in the Control, Block and Block + Arg groups. **(C)** Representative epifluorescence microscopic images of SIRT1 in the Control, Block, and Block + Arg groups. Bar = 50 μm. **(D)** Expression of ZGA genes measured by qRT-PCR. *EIF1A*: eukaryotic translation initiation factor 1A; *DPPA2*: developmental pluripotency associated gene 2; *ZSCAN4*: zinc finger and SCAN domain containing 4. *n* =3; different letters (a, b) indicate significant differences (*P* = 0.05). **(E)** Results of the statistical analysis of embryonic SIRT1 intensity in the Control, Block, and Block + Arg groups. Control: *n* = 40; Block: *n* = 40; Block + Arg: *n* = 40.

To determine the function of arginine, the embryos were cultured in a normal PZM-3 medium (0.12 mM arginine), a modified PZM-3 medium (0 mM arginine) and a modified PZM-3 medium supplemented with arginine (0.12 mM arginine). The zygotes grown in normal PZM-3 medium (Control group) transition to the blastocyst stage at the proper rate, developing to the 4-cell stage at 48 h and the morula at 72 h. Zygotes grown in the modified PZM-3 medium can cleave normally and develop to the 4-cell stage at 48 h, but were blocked at the 4-cell stage at 72 h (called Block group) and could not continue to develop to the blastocyst stages (Figure 3A). The zygotes cultured in modified PZM-3 medium supplemented with arginine (Block + Arg group) were observed to develop to the morula stage within 72 h. The 4-cell stage embryo development block rate at 72 h (Figure 3B) was significantly increased in the Block group compared to the Control group (*P* < 0.05). The rate of 4-cell developmental arrest in the Block + Arg group was significantly decreased than that in the Block group (*P* < 0.05). The results indicate that 4-cell block occurred in the absence of arginine under nutrition restriction, and arginine supplementation reduced 4-cell block under nutrition restriction.

The previous sequencing results confirmed that ZGA in porcine embryos was initiated at the 4-cell stage. As this was precisely the stage at which the embryos arrest in the absence of arginine under nutrition restriction. Failure of ZGA is the cause of the 4-cell block. Therefore, we explored whether arginine was associated with ZGA by detecting the expression of zygotic genome activation marker gene (*EIF1A, DPPA2*, and *ZSCAN4)* and the early transcription product SIRT1 protein of 4-cell embryos in three groups. Compared with the Control group, the mRNA expression of the zygotic genes of 4-cell embryos was significantly decreased in the Block group (Figure 3D). The expression of the zygotic gene and in 4-cell embryos was significantly increased in the Block + Arg group compared to the Block group (*P* < 0.05). Next, the expression of the protein SIRT1 of 4-cell embryos, was detected in the three groups (Figure 3C). The expression of the protein SIRT1 of 4-cell embryos was also significantly decreased in the Block group compared to the Control group (Figure 3E). The expression of the SIRT1 protein in 4-cell embryos was also significantly increased in the Block + Arg group compared to the Block group (*P* < 0.05). Taken together, these observations indicate that arginine promotes ZGA under nutrition restriction in porcine embryos.

### Arginine Affects Embryonic Metabolism During ZGA Under Nutrition Restriction

**Figure 4:**
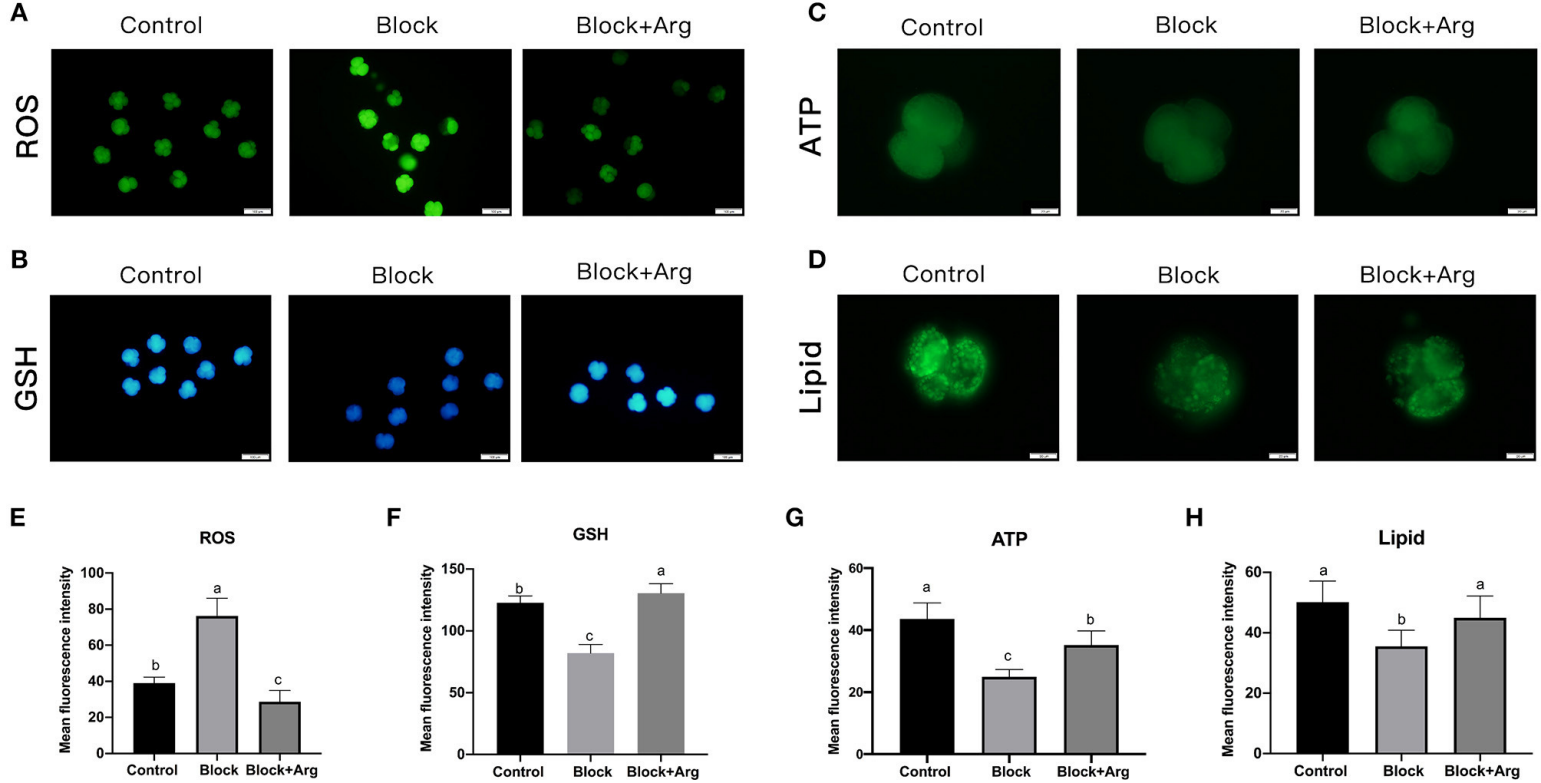
Effects of arginine on the metabolism status of embryos during ZGA under nutrition restriction. **(A)** The 4-cell embryos were stained with DCFH-DA to measure the intracellular levels of ROS in the Control, Block and Block + Arg groups. Bar = 100 μm. **(B)** The 4-cell embryos were stained with Cell Tracker Blue to measure the intracellular levels of GSH in the Control, Block and Block + Arg groups. Bar = 100 μm. **(C)** ATP level of 4-cell embryos stained with BODIPY-ATP in the Control, Block and Block + Arg groups. Bar = 50 μm. **(D)** Images of 4-cell embryos obtained by epifluorescence microscopy. Lipid droplets were stained with the lipophilic dye BODIPY 493/503 in the Control, Block and Block + Arg groups. Bar = 50 μm. **(E–H)** Results of the statistical analysis of embryonic ROS, GSH, ATP and lipid droplet intensity in the Control, Block and Block + Arg groups. Control: *n* = 30; Block: *n* = 30; Block+Arg: *n* = 30. Different letters denote significant differences (*P* < 0.05).

The level of ROS in embryos is related to developmental block and ZGA. Thus, to investigate the underlying changes in cellular ROS, we detected the ROS expression levels of each group (Control group, Block group, and Block + Arg group) by using DCFH-DA (Figure 4A). The ROS content of 4-cell embryos in the Block group was significantly higher than that of embryos in Control group (*P* < 0.05). The ROS level of 4-cell embryos in the Block + Arg group was significantly reduced (*P* < 0.05) compared with that in the Block group (Figure 4E). In contrast, the GSH level (Figure 4B) of 4-cell embryos in the Block + Arg group was significantly increased (*P* < 0.05) compared with that in the Block group (Figure 4F).

Subsequently, we evaluated the content of ATP and lipid droplets in 4-cell embryos treated with BODIPY-ATP (Figure 4C) and BODIPY-LD (Figure 4D). The ATP content of 4-cell embryos was significantly decreased (*P* < 0.05) in the Block group compared to the Control group. However, the ATP content of 4-cell embryos in the Block + Arg group was higher (*P* < 0.05) than that in the Block group (Figure 4G). We detected the lipid droplet content of 4-cell embryos and found that its content was significantly lower in the Block group than in the Control group. The lipid droplet content of 4-cell embryos was also higher in the Block + Arg group (*P* < 0.05) than that in the Block group (Figure 4H).

### Arginine Regulates ZGA Through Promoting Polyamine Synthesis Under Nutrition Restriction

**Figure 5:**
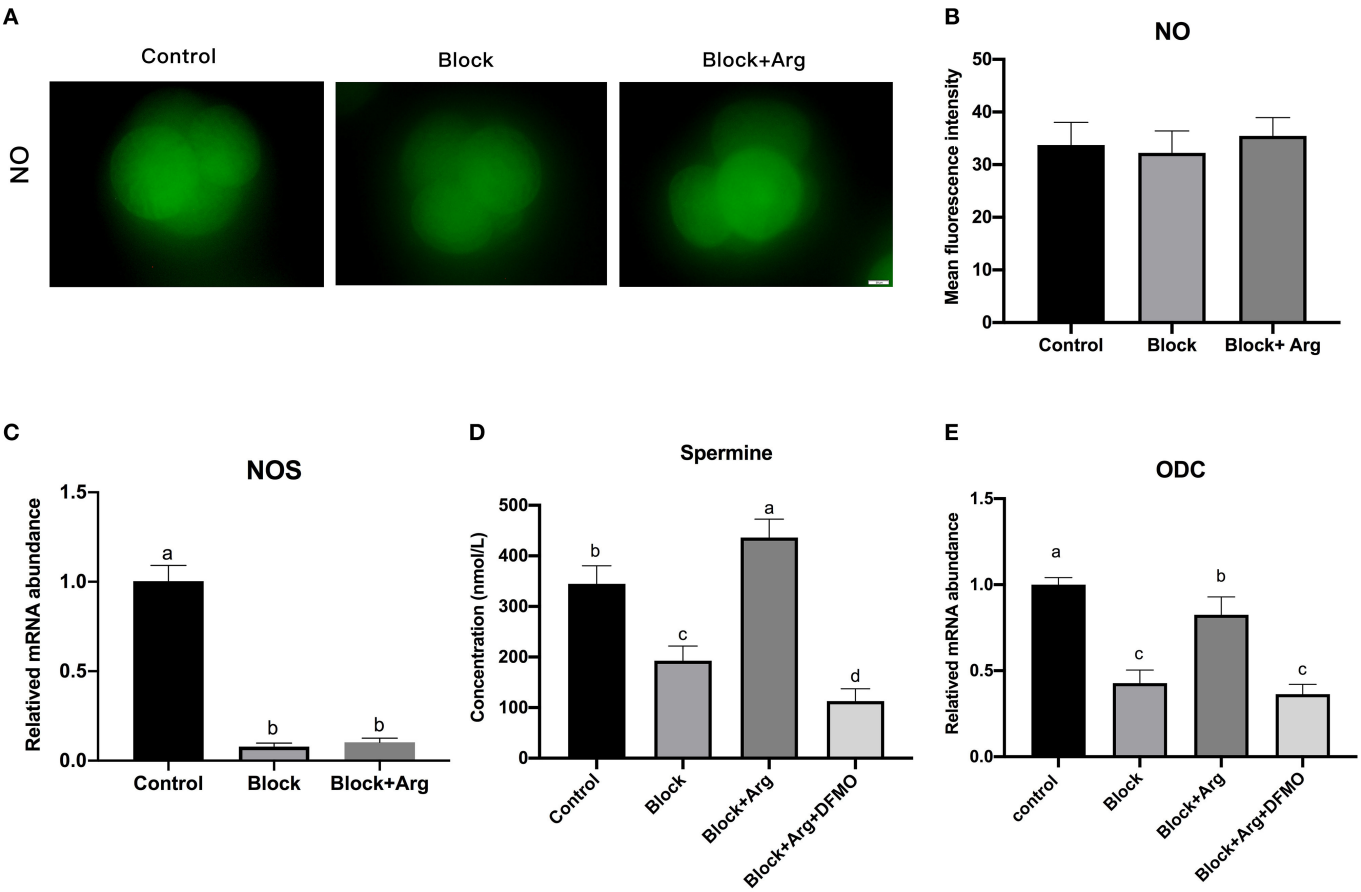
Analysis of the arginine metabolic pathway during ZGA under nutrition restriction. **(A)** Representative images of DAF-FM fluorescence in the Control, Block and Block + Arg groups. Nitric oxide production viewed by using DAF-FM staining of 4-cell embryos. Bar = 50 μm. **(B)** Results of the statistical analysis of embryonic NO intensity in the Control, Block and Block + Arg groups. Control: *n* = 30; Block: *n* = 30; Block + Arg: *n* = 30. **(C)** Expression of the *NOS* gene measured by qRT-PCR. *NOS*: nitric oxide synthase. **(D)** Results of the statistical analysis of spermine content in the culture medium at 48 h after IVC in the Control, Block, Block + Arg and Block + Arg + DFMO groups. Control: *n* = 4; Block: *n* = 4; Block+Arg: *n* = 4; Block + Arg + DFMO: *n* = 4. **(E)** Expression of the *ODC1* gene measured by qRT-PCR. *ODC1*: ornithine decarboxylase 1. *n* =3; different letters (a, b) indicate significant differences (*P* = 0.05).

Arginine can play a role in cells by producing a variety of metabolites through various metabolic pathways. Therefore, we explored the metabolic pathways and metabolites of arginine during zygotic genome activation under nutrition restriction. At 48 h after IVC, we detected the NO content (Figure 5A) of 4-cell embryos in the Control, Block, Block +Arg groups. The NO content of 4-cell embryos in the Block + Arg group was slightly increased compared with that in the Block group, but the difference among the three groups was not significant (*P* > 0.05) (Figure 5B). Next, we detected the *NOS* gene expression of 4-cell embryos in the three groups and found that the *NOS* gene expression in the Block and the Block + Arg groups was significantly decreased (*P* < 0.05) compared with that in Control group, but the difference between the Block and Block + Arg groups was not significant (*P* > 0.05) (Figure 5C).

At 48 h after IVC, the contents of polyamine (agmatine, S-adenosyl-L-methionine, putrescine, cadaverine, spermidine, and spermine) in the embryo culture medium in the Control, Block, and Block +Arg groups were evaluated. It was found that only spermine could be detected, other polyamines were not present in the medium. The spermine content of the culture medium in the Block group was significantly decreased (*P* < 0.05) compared with that in the Control group. The spermine content of the culture medium in the Block + Arg group was significantly increased (*P* < 0.05) compared with that in the Block group (Figure 5D). In addition, the mRNA expression of *ODC1*, the key enzyme of polyamine synthesis, in the Block and the Block + Arg groups was significantly decreased (*P* < 0.05) compared with that in Control group, and the mRNA expression of *ODC1* in the Block + Arg group was significantly increased (*P* < 0.05) compared with that in the Block group (Figure 5E). The above results indicate that arginine may affect ZGA through the polyamine pathway. To test our hypothesis, zygotes were cultured in the modified PZM-3 medium supplemented with DFMO and arginine (Block + Arg + DFMO group). The spermine content of medium (Figure 5D) and the mRNA expression of *ODC1* (Figure 5E) of 4-cell embryos in the Block + Arg + DFMO group were significantly decreased (*P* < 0.05) compared with those in the Block + Arg group.

**Figure 6:**
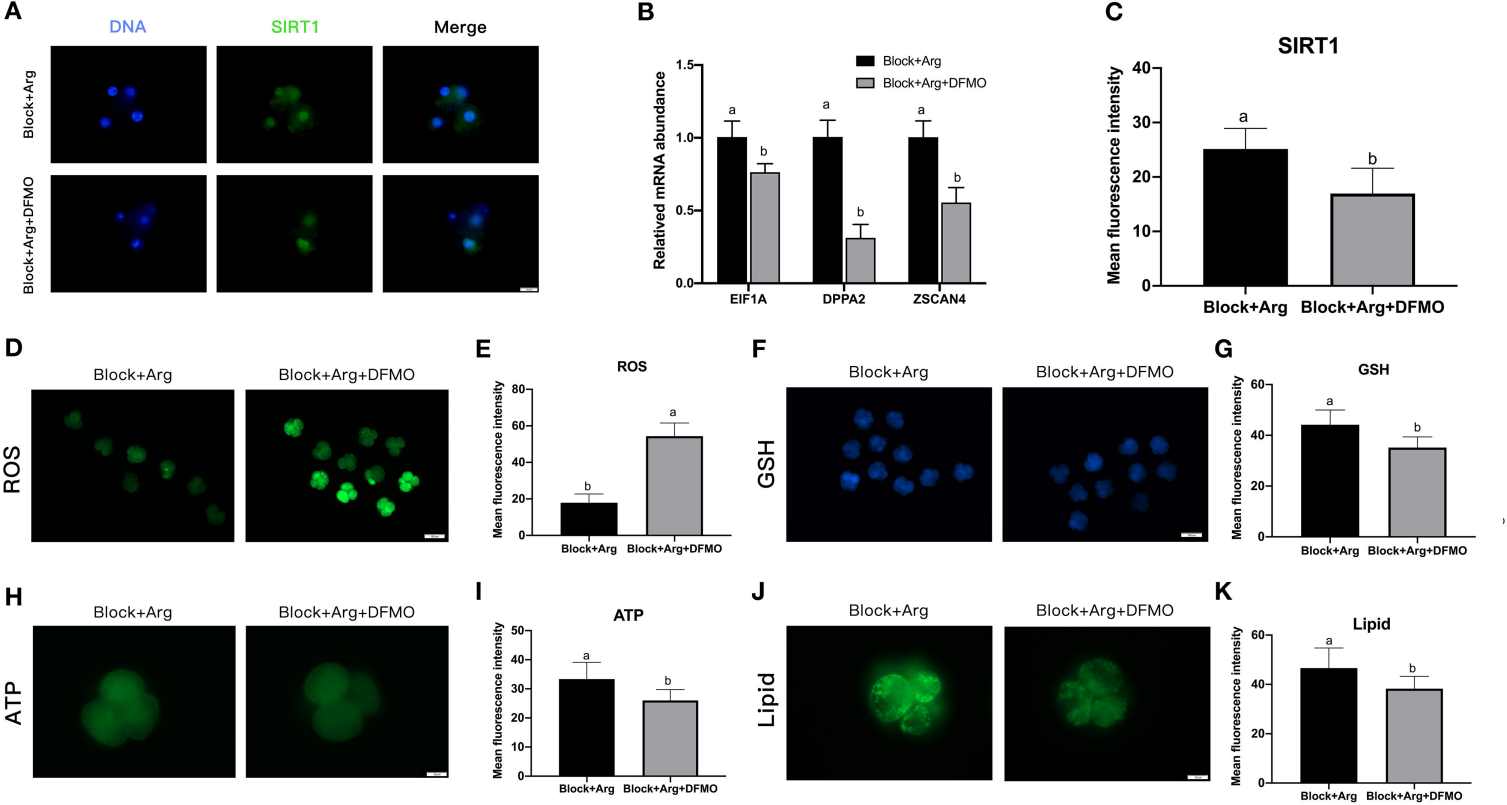
The effect of arginine on ZGA *via* ODC1 under nutrition restriction. **(A)** Representative fluorescence images of SIRT1 in the Block + Arg and Block + Arg + DFMO groups. Bar = 50 μm. **(B)** Expression of ZGA genes measured by qRT-PCR in the Block + Arg and Block + Arg + DFMO groups. *n* =3; different letters (a, b) indicate significant differences (*P* = 0.05). **(C)** Results of the statistical analysis of whole-cell SIRT1 intensity in the Block + Arg and Block + Arg + DFMO groups. Block + Arg: *n* = 30; Block + Arg + DFMO: *n* = 30. **(D)** The 4-cell embryos were stained with DCFH-DA to measure the ROS levels in the Block + Arg and Block + Arg + DFMO groups. Bar = 100 μm. **(E)** Results of the statistical analysis of embryonic ROS intensity in the Block + Arg and Block + Arg + DFMO groups. Block + Arg: *n* = 30; Block + Arg + DFMO: *n* = 30. **(F)** The 4-cell embryos were stained with Cell Tracker Blue to measure the GSH levels in the the Block + Arg and Block + Arg + DFMO groups. Bar = 100 μm. **(G)** Results of the statistical analysis of embryonic GSH intensity in the Block + Arg and Block + Arg + DFMO groups. Block + Arg: *n* = 30; Block + Arg + DFMO: *n* = 30. **(H)** The 4-cell embryos were stained with BODIPY-ATP to measure the ATP levels in the Block + Arg and Block + Arg + DFMO groups. Bar = 50 μm. **(I)** Results of the statistical analysis of embryonic ATP intensity in the Block + Arg and Block + Arg + DFMO groups. Block + Arg: *n* = 30; Block + Arg + DFMO: *n* = 30. **(J)** The 4-cell embryos were stained with the lipophilic dye BODIPY 493/503 to measure Lipid droplets in the Block + Arg and Block + Arg + DFMO groups. Bar = 50 μm. **(K)** Results of the statistical analysis of embryonic Lipid droplets intensity in the Block + Arg and Block + Arg + DFMO groups. Block + Arg: *n* = 30; Block + Arg + DFMO: *n* = 30. Different letters denote significant difference (*P* < 0.05).

The expression levels of SIRT1 protein (Figure 6A) and the zygotic genes (Figure 6B) of 4-cell embryos in the Block + Arg + DFMO group were significantly decreased (*P* < 0.05) compared with those in the Block + Arg group (Figure 6C). It was found that ROS contents in the Block + Arg+ DFMO group were significantly increased (*P* < 0.05) compared with those in the Block + Arg group (Figures 6D,E), whereas the GSH (Figures 6F,G), ATP (Figures 6H,I) and lipid droplet (Figures 6J,K) contents were significantly decreased. In summary, these findings show that arginine may regulate ZGA through promoting polyamine synthesis under nutrition restriction.

## Discussion

In this study, to examine the role of arginine metabolism in early porcine embryos, we collected *in vivo* porcine embryos at different stages and performed single-cell RNA-seq analysis to detect the transcription patterns of genes involved in arginine metabolism during the *in vivo* development of the porcine embryos. The sequencing results revealed that arginine metabolism plays an important role in ZGA. Subsequently, the results of an *in vitro* embryo culture experiment indicated that arginine affects ZGA under metabolic stress. Further analysis showed that arginine affects ZGA under metabolic stress through the polyamine pathway.

Early embryonic development in mammals refers to the process from fertilization to blastocyst implantation in the uterus, which takes 14–18 days in pigs (42). After fertilization, porcine embryos generally need to remain in the oviduct for 2 days. The 4-cell embryo is formed at 48 h after fertilization. The embryos enter the uterus from the oviduct by day 3 after fertilization. The blastocyst is formed by day 5 after fertilization in the oviduct. We collected *in vivo* embryos from each stage following this pig embryonic development program for complete transcriptome sequencing. However, the estrus duration and ovulation period of pigs are long, and there are ~2 days after estrus during which breeding can occur, and differences between individuals are large, so it is difficult to accurately time the collection of embryos at each developmental stage. Therefore, the number of embryos collected was not large, but we strictly performed transcriptome sequencing with more than three biological replicates per stage. The PCA showed good data repeatability for each embryonic stage, validating the rigor and reliability of the results. ZGA begins at different times after fertilization and numbers of cleavages in different animals. The major ZGA occurs at the 2-cell stage in mouse embryos, at the 4–8 cell stage in human embryos, and at the 8-cell stage in bovine embryos (43). The most obvious manifestation of ZGA is an enormous growth in transcript level (44). Differentially expressed gene analysis showed that the number of differentially upregulated genes was the largest in the comparison of the 4-cell stage with the 2-cell stage, indicating that ZGA occurs at the 4-cell stage. This result is consistent with previous reports (45–47).

DEG analysis also revealed a number of activated genes and provided strong data support for the further study of the genome activation mechanism. Several genes have been reported as marker genes and potential regulators of ZGA in pigs. For example*, EIF1A* is used as an endogenous marker gene for mouse ZGA. *EIF1A* can be detected at the zygote stage and is expressed in the 2-cell stage (48). This gene has been used successfully as a marker for ZGA in other species; for example, in bovine (49) and human embryos (50); moreover, *EIF1A* is increased at the eight-cell stage. *EIF1A* is also a marker gene of porcine ZGA (51), and we found that *EIF1A* is highly expressed at the 4-cell stage. It was similarly evaluated as a marker gene for ZGA in our subsequent experiment and in another study (52). Another marker, *DPPA2*, acts as the main activator of the ZGA transcription program by directly regulating the mouse ZGA transcription factor Dux (53). *DPPA2* contributes to the transcriptional regulation of ZGA in humans and mice and is significantly activated in mouse 2-cell embryos and 4–8-cell embryos (54). Our study indicated that *DPPA2* is highly expressed at the 4-cell stage and continues to be highly expressed at the 8-cell and morula stages. *DPPA2* may play the same role in pig embryonic development as in human and mouse embryos. *DPPA2* was used as the key zygotic gene in previous studies in pigs (55). However, the detailed function of *DPPA2* in pig embryonic development requires further study. *ZSCAN4* is well known as a marker of ZGA in humans (56) and mice (57). The role of *ZSCAN4* in early embryonic development in cattle (58) and sheep (59) has also been reported. However, there is no clear data regarding *ZSCAN4* in pig ZGA. Our study showed that *ZSCAN4* was expressed at the 4-cell stage. Interestingly, *ZSCAN4* is specifically expressed at the 4-cell stage and is hardly expressed at other developmental stages. Its correlation with arginine metabolism-related genes was similar to that of *EIF1A* and *DPPA2*. The level of DNA methylation decreases when zygotic genomic activation occurs, which is catalyzed by DNA methyltransferases (Dnmts). *DNMT1* serves as the major maintenance methyltransferase. Our results showed that *DNMT1* expression was decreased at the 4-cell stage compared to the zygote stage. However, the expression level of *DNMT1* in early pig embryos is much lower than that of the *EIF1A* gene. Additionally, the SIRT1 protein has been used as a marker protein for mouse and porcine ZGA since it is an early product of transcription (18, 52). Elevated expression of SIRT1 protein in porcine zygotes promotes blastocyst formation (60). Therefore, we selected the *EIF1A, DPPA2* and *ZSCAN4* genes and SIRT1 protein as markers to evaluate the occurrence of porcine ZGA.

Early mammalian development is a complex and orderly process, with the proliferation and differentiation of embryos, the dynamic changes of gene regulatory networks, the erasure and re-establishment of epigenetic modifications, and the metabolic level from simple resting to active and complex. More and more studies have found that metabolism plays an important regulatory role during embryonic development. Our study initially explored the metabolic changes in porcine ZGA through transcriptome analysis and found that in addition to arginine metabolism, involves oxidative phosphorylation, amino acid biosynthesis, the TCA cycle, pyruvate metabolism and proline metabolism. Studies in humans and mice have also found that pyruvate is an essential nutrient for development during and after ZGA in mouse and human embryos. Pyruvate is essential for nuclear localization, and the failure of TCA cycle enzymes to enter the nucleus is associated with the loss of specific histones and ZGA blockade, demonstrating that pyruvate is necessary for the subsequent development to proceed smoothly (18). This result is consistent with our findings. However, the role of the TCA cycle in early embryonic development of the pig should be further verified. Interestingly, many pathways, such as amino acid biosynthesis and fatty acid metabolism, are active at the 4-cell stage (ZGA stage) but not at the 2-cell and zygote stages, which indicates that these metabolic pathways and the metabolites that they produce are critical to genome activation. A study shown that fatty acid metabolism was revealed to be an indicator for the maternal-to-zygotic transition in porcine *in vitro* fertilization (IVF) embryos (61). One missing component of mapping techniques applied to embryonic development is metabolomics, as current metabolomics and metabolite flow studies remain challenging for a small number of cells. The latest study broke through the technical bottleneck of early embryo metabolomics determination, and studied the metabolomics and transcriptomics for the first time to systematically map the metabolic remodeling and metabolic regulation in mouse 2-cell stage embryos and blastocyst samples in early embryo development. Comprehensive metabolomic and transcriptomic analysis revealed that 2-cell (ZGA stage) embryos mainly metabolized methionine, polyamines, and glutathione and remained in a reduced state, but blastocysts were mainly in an oxidized state (61). It is worth noting that metabolomic analysis cannot directly indicate the activity of metabolic pathways, and isotopic tracing analysis of embryos deserves further study. The important reason why we did not study the effect of arginine on zygotic genome activation by removing arginine in normal culture conditions is that arginine family amino acids can be converted into arginine, which cannot achieve the purpose of completely removing arginine. In the future, if isotope labeling can be applied, it will be possible to trace the metabolic pathway and solve our problem.

When the porcine oocyte is discharged from the follicle, it stores more yolk for its own nutrition, but with the acceleration of embryonic development, the stored yolk cannot meet the needs of embryonic development, so nutrients must be obtained from the environment. The embryo obtains nutrients from the oviduct fluid and uterine fluid in mammals and from the culture medium in an *in vitro* culture environment. Early embryos produced *via* IVF often stagnate at a certain stage, a phenomenon called developmental arrest. Therefore, we removed nutrients from the medium such that the embryos could develop to the 4-cell stage using maternally derived substances, but development was arrested thereafter. Abnormal ZGA is one of the main causes of early embryonic developmental arrest in mammals (62, 63). The time of mammalian developmental arrest is consistent with the time of ZGA, and developmental arrest in pigs occurs at the 4-cell stage (64). Embryos in the Block group were cultured in the absence of nutrients, and most embryos could cleave to 4-cell arrest, due to the inability of ZGA. The mouse embryos cultured in a modified KSOM medium that is devoid of all amino acids, proteins and pyruvate are arrested at the 2-cell stage. However, in a study, porcine embryos grown in modified PZM-5 medium (without amino acids, hypotaurine, and pyruvate) developed into blastocysts. This study used porcine parthenogenetically activated embryos, which were first cultured in normal PZM-5 medium for 24 h after activation and then transferred to modified PZM-5 medium (20). In our study, the IVF embryos were directly transferred to the modified PZM-3 (without amino acids, hypotaurine and pyruvate) after 6 h of fertilization. The specific reasons need further experimental analysis. The expression levels of zygotic genome activation marker genes EIF1A, DPPA2, ZSCAN4, and SIRT1 protein of 4-cell embryos in the Block group were significantly decreased than those of the Control group. This indicates that embryos cultured in the modified PZM-3 medium failed to initiate ZGA due to metabolic stress. Arginine can promote ZGA under metabolic stress. Study reported that pyruvate is required for ZGA. Furthermore, we found that a-KG added to the modified KSOM medium supports development to the blastocyst stage and completely rescues the 2-cell block. Interestingly, only proline and arginine among the non-essential amino acids that can generate a-KG (arginine, proline, glutamine, glutamic acid, alanine and glutamine, alanine, cysteine, serine, glycine) can rescue the 2-cell block. Neither alanine nor serine, which can generate pyruvate, can rescue the failure of ZGA (18). This finding is consistent with our results. However, the role of other amino acids except arginine on porcine ZGA needs to be further studied.

In general, basal ROS production can serve as a natural regulatory mechanism of cells involved in the regulation of the general redox state (65). However, prolonged excessive ROS production can lead to oxidative stress, so ROS production must be kept to a minimum. Among the various deleterious factors that may lead to developmental arrest, the effects caused by excess ROS are considered to be the most significant (66). Previous studies have reported that early embryo developmental arrest is associated with increased ROS levels (67). Icariin could decrease ROS levels and modulate the expression of the ZGA marker gene eIF1A, thereby promoting the development of H_2_O_2_-pretreated mouse pre-implantation embryos (66). Melatonin relieves two-cell block *via* the non-receptor pathway in mice (68). Our research results show that ROS levels increase when ZGA fails, and the addition of arginine can reduce ROS levels and improve GSH levels in 4-cell embryos and initiate ZGA. Arginine can exert antioxidant and anti-inflammatory functions *via* ROS (69). Our results indicate that arginine can increase the ATP level of the embryo. Early cleavage stage embryos in mammals rely on oxidative phosphorylation of directly available energy substrates such as pyruvate, fatty acids, and amino acids to generate ATP (70). However, an energy imbalance leads to developmental arrest in diverse animal species (71). Pig embryos in particular contain numerous large LDs as well as large amounts of intercellular lipid bilayers compared to other species. Endogenous fatty acids are stored in the lipid droplets of the embryo (72). Fatty acid metabolism may be an indicator of MZT in porcine IVF embryos. Lipid droplets are organelles that are specialized for fat storage but are also closely related to the endoplasmic reticulum, mitochondria and other organelles, and they can regulate cell metabolism (73). Interestingly, our results show that the lipid droplet content in 4-cell embryos is reduced when ZGA is blocked, whereas arginine addition increases the lipid droplet content in 4-cell embryos. It also suggests that lipid droplets in porcine embryos may be important when embryos under metabolic stress.

The results of this study show that inhibiting the expression of *ODC1* can cause arginine to lose its effect on ZGA. In the polyamine biosynthetic pathway, *ODC1*, the first and rate-limiting enzyme, catalyzes the decarboxylation of L-ornithine, leading to the formation of polyamine. Our sequencing results indicated that *ODC1* was highly expressed at the 4-cell stage. Polyamines also promote higher transcription of *ODC1* in 2-cell embryos of mice, and *ODC1* expression increases throughout the blastocyst stages (74). Polyamines can stimulate the transcription of translation factors (75). For example, polyamines can regulate translation *via* the expression of eukaryotic translation initiation factor 5A (*EIF5A*) (76). We found that *ODC1* is related to the expression of *EIF1A*, and inhibiting the expression of *ODC1* reduced the expression of the *EIF1A* gene. Ultrastructural analysis has also revealed a possible role of polyamines in nucleolar formation. The formation of nucleoli is the main marker of porcine ZGA. This may be the main reason that arginine can generate polyamines through *ODC1* and affect ZGA.

Zygotic genome activation is an essential process for embryogenesis. A better understanding of its internal regulation mechanisms would have important value for both basic research and practical applications. Our study found a potential role for arginine metabolism in this process, which may help to develop new ideas for exploring the regulatory mechanisms of ZGA.

## Conclusions

In conclusion, arginine metabolism-related genes are highly expressed during ZGA by single-cell transcriptome analysis. Arginine promotes ZGA to reduce 4-cell block under nutrient deprivation conditions. Subsequently, arginine can reduce oxidative stress and enhance energy levels for maintaining ZGA. Arginine may generate polyamine for ZGA in porcine embryos under nutrition restriction conditions.

## Data Availability Statement

The datasets presented in this study can be found in online repositories. The names of the repository/repositories and accession number(s) can be found in the article/supplementary material.

## Ethics Statement

The animal study was reviewed and approved by the Animal Care and Use Committee of the Key Laboratory of Animal Production, Product Quality and Security, Ministry of Education.

## Author Contributions

DC, RH, and TZ conceived and designed the experiments. TZ, YZhe, and TK performed experiments including scRNA-Seq and embryo manipulation. HW and YZha performed bioinformatics analysis. LY and HJ supervised and provided continuous guidance for the experiment. TZ wrote the paper. All authors reviewed the manuscript. All authors contributed to the article and approved the submitted version.

## Funding

This study was supported by the National High Technology Research and Development Program of China (2013AA102503) and the National Natural Science Foundation General Program of China (32072747).

## Conflict of Interest

The authors declare that the research was conducted in the absence of any commercial or financial relationships that could be construed as a potential conflict of interest.

## Publisher's Note

All claims expressed in this article are solely those of the authors and do not necessarily represent those of their affiliated organizations, or those of the publisher, the editors and the reviewers. Any product that may be evaluated in this article, or claim that may be made by its manufacturer, is not guaranteed or endorsed by the publisher.
